# Pixel-based versus object-based identification of scenic resources using Gaofen-2 images: A case study of Yesanpo National Park

**DOI:** 10.1371/journal.pone.0267435

**Published:** 2022-04-28

**Authors:** Zhe Jia, Anchen Qin

**Affiliations:** College of Landscape Architecture and Tourism, Hebei Agricultural University, Baoding, China; Duy Tan University, VIET NAM

## Abstract

Scenic resources can serve as symbols of a region’s natural resources and culture and are often the stimulus for the development of national parks. Thus, careful scientific planning and effective management based on the identification and evaluation of scenic resources are key for the sustainable development of national parks. In this study, one object-oriented and three pixel-based (maximum likelihood classification, neural network, and support vector machine) classification methods were applied to identify scenic resources in Yesanpo National Park using high-resolution Gaofen-2 images. The classification accuracy of these scenic resources was evaluated through systematic sampling, which improved the objectivity and accuracy of the classification precision evaluation. All methods met the precision requirements of scenic resource identification, and the accuracy of object-oriented classification was the highest. The application scope of the different methods varies, and suitability can be determined according to the needs of scenic resource recognition. Collectively, this study has proposed an effective and practical method for the identification of scenic resources within Yesanpo National Park, which is of significance for its future planning and management. Moreover, this strategy can be applied by other national park planners to select areas for tourism development, formulate sustainable development strategies, and provide technical support and decision-making guidance for national park planning and management.

## Introduction

National parks have ornamental, cultural, and scientific value. The natural and cultural landscapes in these areas are relatively concentrated, thereby allowing people to readily frequent the areas to conduct scientific, recreational, and cultural activities. Moreover, national parks have an important role in protecting ecology, biodiversity, natural environment, and cultural heritage, while also contributing to the development of tourism, carrying out of scientific research and cultural education activities, and promoting the sustainable development of local economy and society [1, 2]. Indeed, leisure tourism and cultural tourism within national parks represent the fastest growing types of tourism. As such, strategic planning and tourism development have become the focus of research related to national parks. Specifically, the accurate identification and characterization of scenic resources have become necessary for scientific planning and reasonable development.

Owing to the diversity and complexity of scenic resources, the traditional methods of field manual survey and resource identification are susceptible to the difficulties of terrain, weather, accessibility, and other factors; furthermore, such surveys are time-consuming and costly, requiring a significant amount of material, human, and financial resources. Hence, it is difficult to comprehensively and efficiently survey national parks [3], resulting in the limited availability of detailed and comprehensive scenic resource data for planning [4]. As a promising solution, the rapid development of remote sensing (RS) technology provides conditions for conducting rapid, multi-scale, and high-precision investigations and the identification of scenic resources; in particular, high-resolution RS images have the advantages of being rich in spatial information and displaying obvious textural features and clearer feature contours, and can be collected through real-time data acquisition [5, 6]. Such imagery is mainly used in forestry, agriculture, urban and rural planning, and environmental research [7]; they are an important source of data for these fields [8] and can be used for plant classification [9], building identification, and landscape evaluation, among other uses. Moreover, they have unique advantages for use in the spatial identification of scenic resources [10–15] based on typical pixel-based classification methods including the maximum likelihood classification (MLC), support vector machine (SVM), and neural network (NN) methods. For example, Yang et al. [16] used the SVM method with SPOT satellite images to classify different landforms in Zhangjiajie and achieved better results than with other classification methods. In other research, based on SPOT-5 images of Zhongshan Cemetery, the methods of MLC, minimum distance, and Mahalanobis distance were used for classification, as well as a method based on the combination of decision tree and Mahalanobis distance, and it was found that the combination method has higher accuracy for the classification of forest resources [17]. In another study, Yuan [18] attempted to extract classification vector maps based on two RS images, SPOT-5 and Quickbird, according to the composition, shape, diversity, and fragmentation of the landscape of Zhongshan Cemetery. The shape and attribute of the surface features extracted using the object-oriented (object-based) classification are highly consistent with the actual surface features. However, past research has concentrated on the identification of scenic resources from high-resolution RS images obtained with SPOT, Quickbird, and Worldview.

Launched in 2014, the Gaofen-2 (GF-2) satellite has a multispectral resolution of up to 3.2 m and panchromatic resolution of up to 0.8 m, and its launch marked the entry of high-resolution Chinese RS satellites into the sub-meter class era [19]. Currently, GF-2 satellite imagery is mainly used in forestry [20–22], urban and rural planning [23–25], land and transportation, and similar disciplines. GF-2 images can also be applied to resource exploration and planning and identification, as well as for monitoring in water conservancy and investigating flooding and earthquake hazards [26], which greatly promotes scientific research and modernization. Although GF-2 images have broad application prospects for the identification of scenic resources that require high spatial resolution, few studies have applied this method to scenic resource recognition.

With the continuous development of RS and computer technology, the classification methods of high-resolution RS images have progressed, among which pixel-based and object-oriented methods are the most representative [27–31]. However, these methods have limited applications [32, 33]; therefore, classification methods should be selected based on the circumstances of the actual situation, and the optimal identification methods should be selected according to the types of RS images and characteristics of recognition objects [34]. However, few comparative studies have investigated the accuracy of using different classification methods to identify scenic resources in GF-2.

Thus, substantial research has been performed to identify individual scenic resources, such as forests, vegetation, and landforms, whereas there is a dearth of research on the identification and evaluation of scenic resource systems. Specifically, no study has reported the construction and application of a scenic resource identification and evaluation system based on GF-2 images combined with 3S technology. This also reflects the limitations of the current research and application of the scenic resource identification system. In fact, researchers, planners, and management personnel often fail to conduct comprehensive and detailed investigations on national parks and instead conduct fieldwork on only scenic resources within certain areas of the national park. Moreover, the use of manual surveys, document collection, and manual visual recognition to guide the planning, protection, and development of scenic resources will inevitably have challenges, including insufficient realism and poor operability. Therefore, the development of strategies that facilitate the comprehensive, efficient, and accurate identification of scenic resources has become increasingly important.

In this study, the Yesanpo National Park was selected as the study area and GF-2 images were used as the data source to determine the classification method most suitable for identifying scenic resources. eCognition was then employed to determine the optimal segmentation scale of GF-2 images based on the characteristics of the image and the scenic resources to be identified. The decision tree algorithm was used to perform object-oriented classification, determine the identification rules and feature parameter combinations, and identify the scenic resources. Additionally, pixel-based MLC, SVM, and NN classification methods were applied to identify scenic resources. The systematic sampling points were then used to calculate the confusion matrix and kappa coefficient to evaluate the classification accuracy of the four classification methods. Finally, the applicable scope of object-oriented and pixel-based classification methods was assessed to determine which method was most suitable for the identification of scenic resources in GF-2 images. These findings will guide natural national park personnel in the efficient and accurate identification of scenic resources to facilitate the selection of suitable areas for the development of leisure tourism and cultural tourism, promote the scientific protection and development of scenic resources, formulate sustainable development strategies for the national park, and improve regional competitiveness.

This study used global positioning system field measurements, RS image recognition, and geographic information system (GIS) spatial analysis, along with other technical means and research methods based on multiple data sources (GF-2 images, forest resource planning, and design survey data), to effectively establish a multi-platform approach of identifying and evaluating scenic resources that is technically difficult and highly comprehensive. Currently, there is minimal research in the field of scenic resources; therefore, the application of this study provides a theoretical basis and sound technical support for the systematic, high-precision, digital identification and evaluation of scenic resources.

## Materials and methods

### Study area

Yesanpo National Park covers a total area of 505.48 km^2^ and is located in the northwest region of Laishui County, near Baoding City, Hebei Province, China, bordering the Fangshan District, Beijing. Located in the deep mountain region at the eastern foot of the northern Taihang Mountains and southern foot of the western Yanshan Mountains, the park is designated as a world geopark, National Park of China, National AAAAA-level Tourist Area, National Forest Park, and National Ecological Tourism Demonstration Area. The park is located on the “step” of the North China Plain, leading up to the Shanxi Plateau, comprising a unique combination of geological features including an alluvial valley, granite fracture structures, a waterfall and canyon, and a karst-cave spring landscape, collectively formed under the action of external geological forces [35, 36]. Yesanpo was one of the first national parks in China to develop tourism. Its scenic resources are highly typical and representative of Northern China and have driven the economic development of the national park and the surrounding areas since the development of tourism in this area after 1986. However, the contradiction between development and protection of the scenic resources has become increasingly prominent with the rapid increase of socio-economic development; therefore, it is crucial to identify the quantity, distribution, scale, and combination of scenic resources in the park accurately and comprehensively, as well as formulate a basis for reasonable and effective scenic area planning and promote the sustainable use of the scenic resources in the area.

According to relevant data from the national park, three typical landform units in the area were selected as representative study sites: the Baili Gorge, an erosional, alluvial landform in the Roach Valley; Longmen Tianguan, a granite fracture structure canyon landform; and Yugu Cave, a karst-cave spring landform [37]. Within the national park, the Baili Gorge is located in the southwest with a total area of 154.0 km^2^, the Longmen Tianguan is located in the northwest with a total area of 66.2 km^2^, and the Yugu Cave is located in the middle with a total area of 57.5 km^2^.

### Data preprocessing

**Fig 1:**
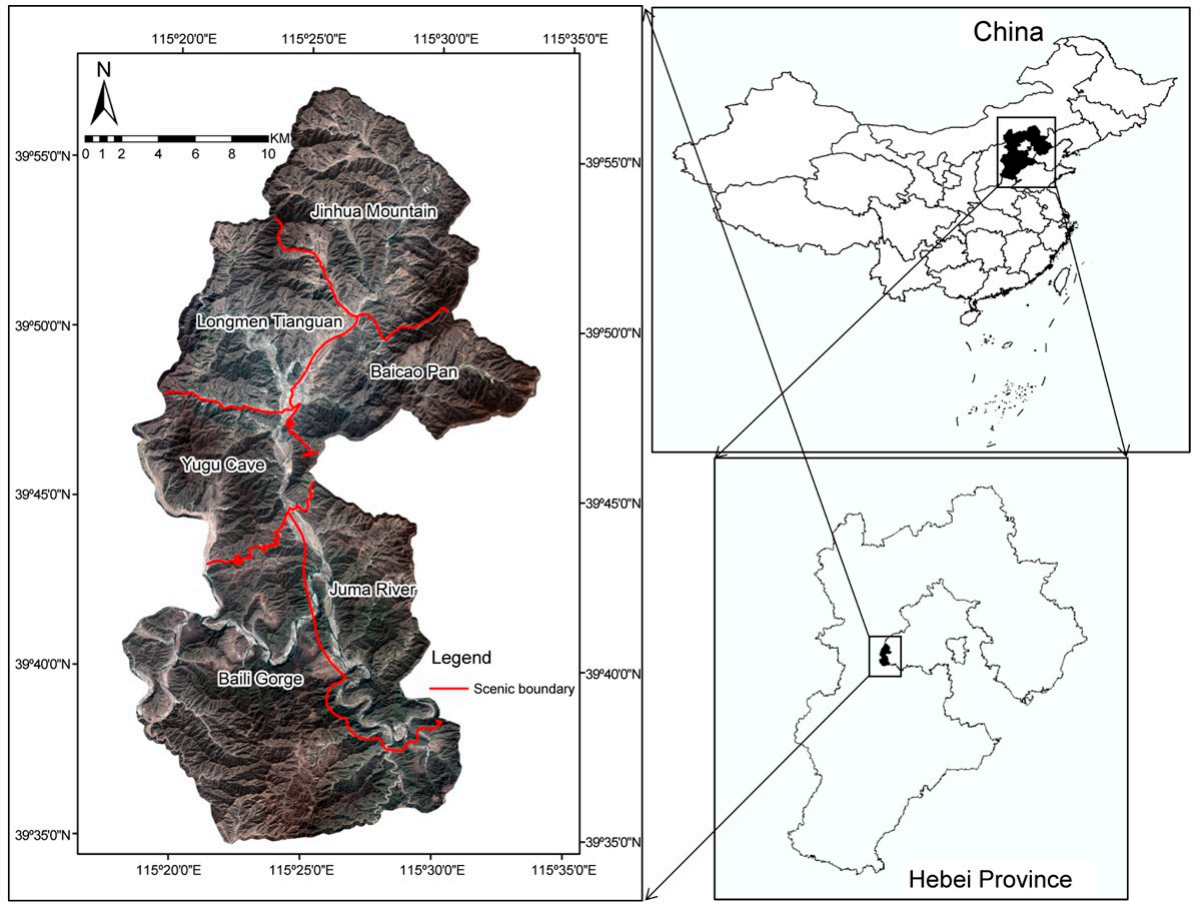
RS image of Yesanpo National Park (Republished from [https://directory.eoportal.org/web/eoportal/satellite-missions/g/gaofen-2] under a CC BY license, with permission from [Hebei Zhongke Sino Star Information Technology Co., Ltd], original copyright [2018]).

GF-2 image data taken on April 16, 2018 from Yesanpo National Park were purchased based on the needs of the study. Using ENVI and ArcGIS to preprocess RS image data, such as ortho-correction, fusion processing (Gram-Schmidt method), geometric correction, and mosaic cropping [38], we eliminated the errors of the images and improved the initial accuracy and resolution. Additionally, ortho-correction was used to address obvious geometric distortions caused by terrain, camera geometry, and sensor-related errors. Geometric correction was then used to eliminate the geometric distortion in the image and produce a new image that meets the requirements of a certain map projection or graphic expression, as shown in Fig 1. Through visual interpretation and field investigation, we obtained a priori knowledge of the characteristics of scenic resources in certain sample areas in GF-2 images, which guided our selection of a certain number of samples for each category. According to the Jeffries-Matusita test results, the sample separation degree in GF-2 image was 1.8030–2.0000, which is greater than 1.8, indicating that the samples have significant differences and good separability and can be used as training samples for dividing scenic resources.

### Object-oriented method for scenic resource recognition

#### Selection of the optimal segmentation scale

Object-oriented scenic resource classification uses eCognition software as the platform to classify GF-2 images, the basis and key technology of which is RS image data segmentation. For this, the selection and estimation of segmentation parameters are crucial to determine the maximum degree of heterogeneity of RS image data segmentation in the national park; furthermore, these parameters also relate to the accuracy of the digital identification of scenic resources. Therefore, the selection of segmentation parameters must consider both the spatial resolution of the GF-2 image data and attributes of scenic resources in the image. Otherwise, an excessively large segmentation scale may easily produce an over-segmentation phenomenon and low classification accuracy; likewise, a segmentation scale that is too small may lead to broken image segmentation, longer running times, and lower efficiency [39]. Therefore, determining the optimal segmentation scale for GF-2 image data for the national park is a priority in research on object-oriented classification.

**Fig 2:**
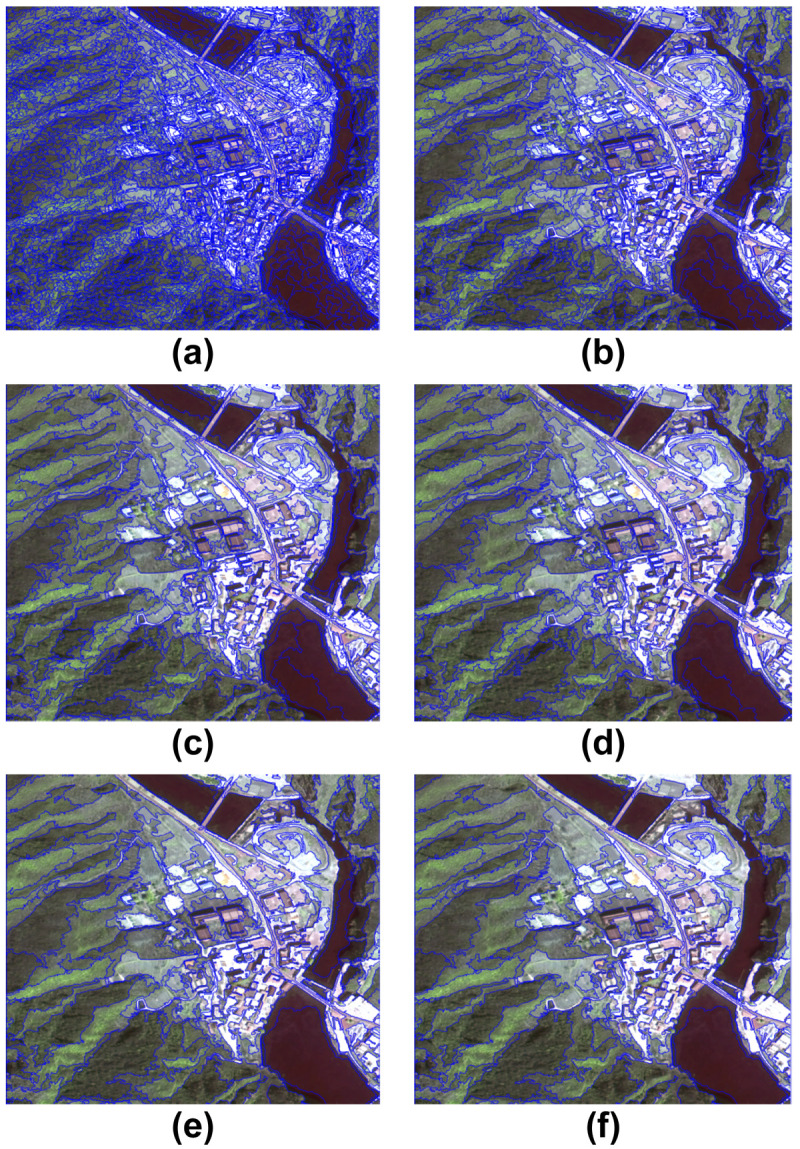
RS images at different segmentation scales. (a) 20; (b) 50; (c) 100; (d) 120; (e) 150; and (f) 200 (Republished from [https://directory.eoportal.org/web/eoportal/satellite-missions/g/gaofen-2] under a CC BY license, with permission from [Hebei Zhongke Sino Star Information Technology Co., Ltd], original copyright [2018]).

Based on the spatial resolution of GF-2 image data and attributes of five types of scenic resources (forest, grassland/shrub, farmland, architecture, and water), to select the best segmentation parameters, several experiments on the segmentation scale parameters were conducted. Experiments with segmentation effects of different parameters and six typical segmentation scales between 20 and 200 (20, 50, 100, 120, 150, and 200) were selected for comparison (shape = 0.1, compactness = 0.5). The effect maps of the area considering multiple types of scenic resources in the national park at different scales are shown in Fig 2.

By analyzing Fig 2, it can be seen that the segmentation effect varies with segmentation scale. The following observations were noted:

1. At a segmentation scale of 20 (Fig 2A), the fragmentation of image segmentation is too high, producing too many segmented objects; the scenic resources are decomposed into multiple polygons, such that only some objects are segmented clearly; the shapes are regular; and the boundaries between scenic resources are obvious.
2. At a segmentation scale of 50 (Fig 2B), there were much fewer segmented image objects than on the previous scale. Larger scenic resources such as forest, grassland/shrub, and architecture are segmented into multiple polygons, and images of forest and grassland/shrub are more fragmented, yet also more clearly demarcated from architecture and waters.
3. At a segmentation scale of 100 (Fig 2C), the quality of the segmentation of forest, grassland/shrub, farmland, architecture, and water is good, with clear plant and non-plant boundaries, that is, clear boundaries between forest, grassland, shrub, farmland, and architecture, and the image exhibits more complete segmentation of waters and roads, with high efficiency in terms of identifying the five scenic resources.
4. At the division scales between 120–200 (Fig 2D–2F), over-segmentation occurs, especially when the boundaries between forest, grassland/shrub, and farmland are not obvious, some of which are divided into whole blocks and easily confused with each other.

Based on the object-oriented segmentation effect evaluation method proposed by Corcoran [40] and Zhang [41], the unsupervised segmentation evaluation method based on the heterogeneity measure was used to evaluate the segmentation effect of GF-2. When the segmentation scale is 100, the minimum value is 0.8416, indicating a superior segmentation effect. In summary, upon comparing the segmentation results of GF-2 images of the national park, the optimal segmentation effect was selected at the following settings: segmentation scale of 100, shape = 0.1, and compactness = 0.5. These settings were determined suitable for the identification and extraction of five types of scenic resources in the national park for this study: forest, grassland/shrub, farmland, architecture, and water.

#### Classification of image data based on optimal segmentation scale

**Fig 3:**
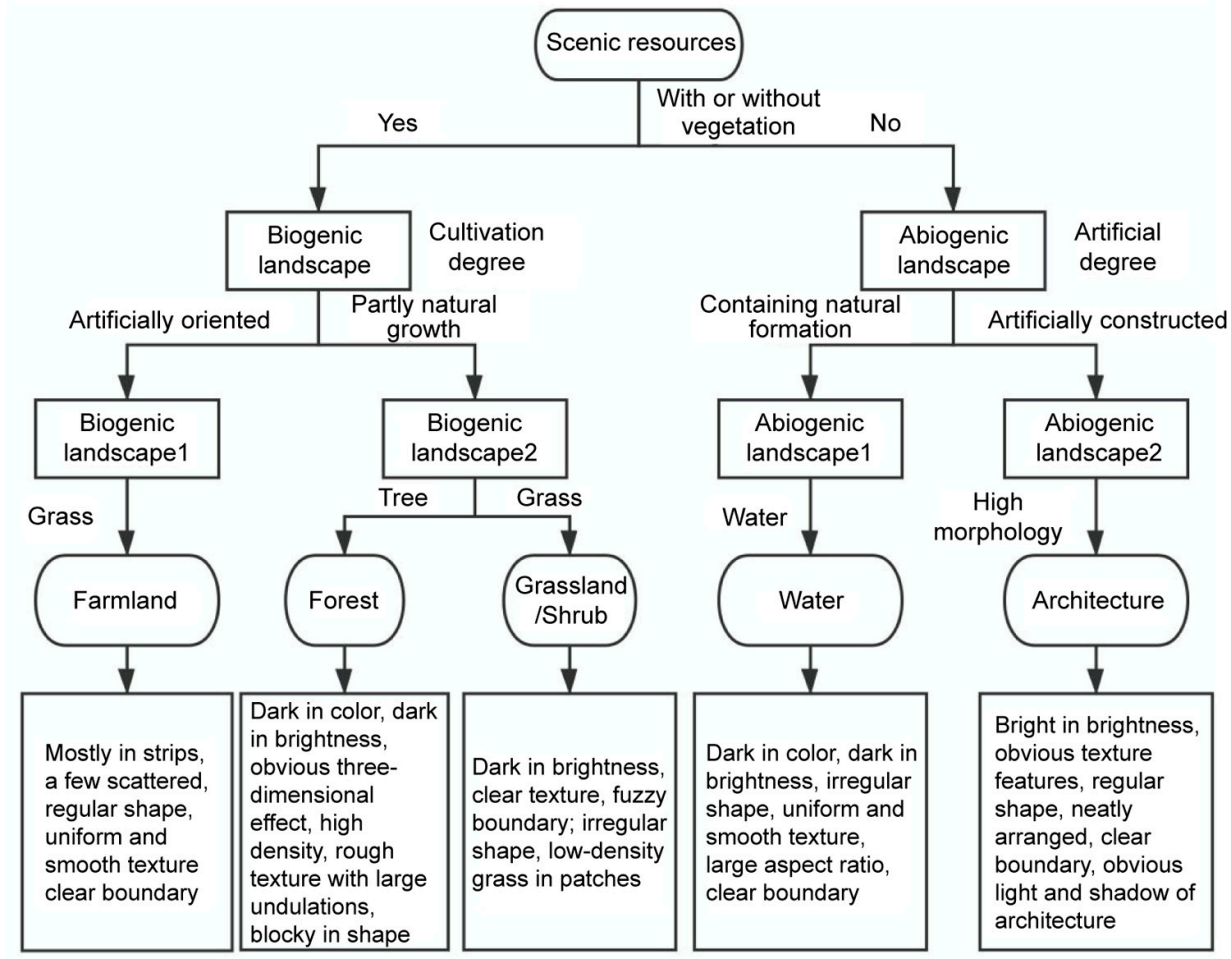
Chart of identification rules of scenic resources.

The decision tree algorithm was used for object-oriented classification of the national park to establish recognition rules to decompose the attributes of different scenic resources in a step-wise manner. By analyzing and comparing the data of each waveband of the GF-2 images, the recognition rules were constructed by analyzing the relationship between the attributes of the five types of scenic resources in the scenic resource identification and evaluation system, as shown in Fig 3.

**Fig 4:**
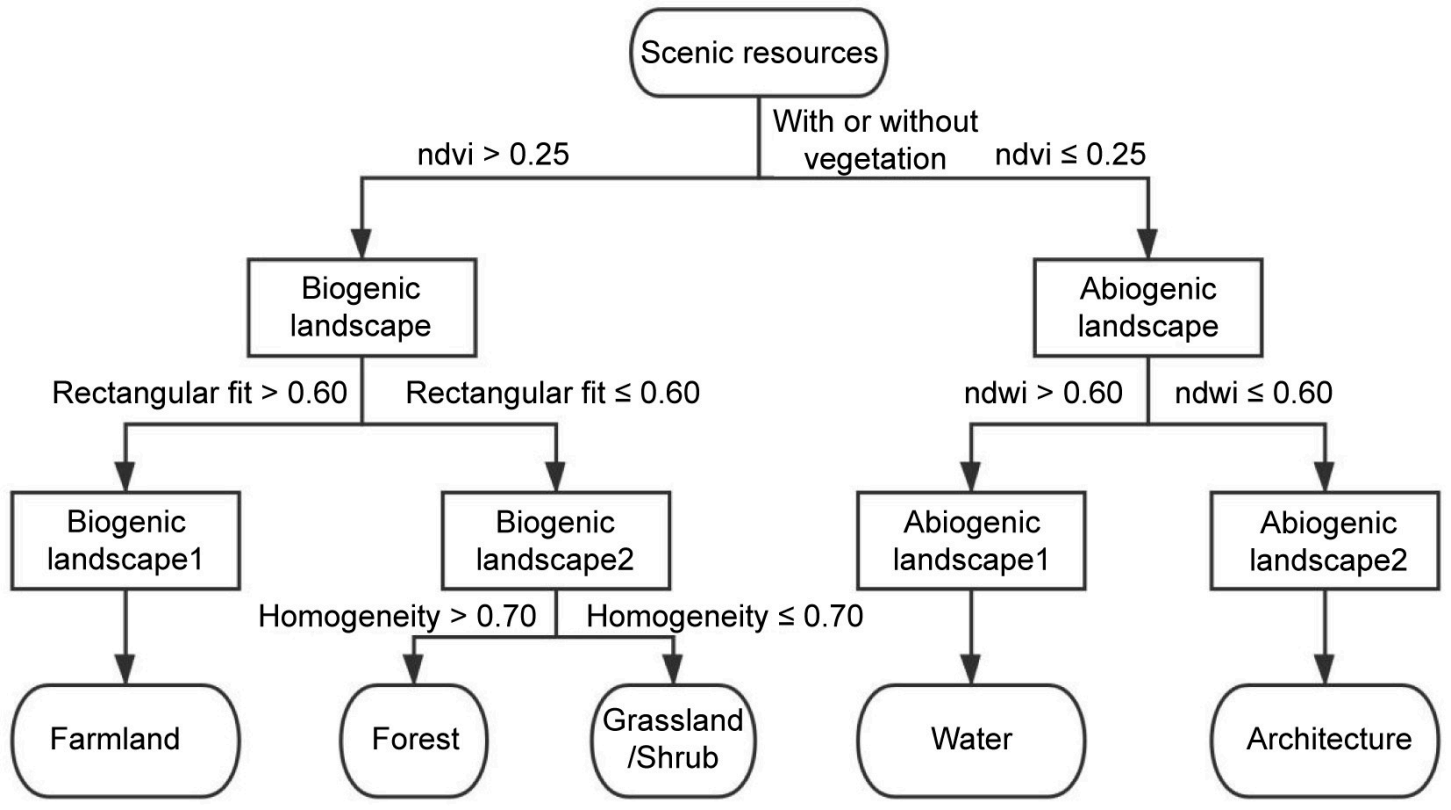
Combination of feature parameters for scenic resource identification in the national park.

The classification features were initially determined according to the recognition rules, and the results are shown in Table 1. On this basis, a combination of feature parameters was then determined, as shown in Fig 4.

**Table 1:** Initial classification characteristics of scenic resources.

Identifying information	Features
**Water**	No vegetation, abiogenic landscape, containing natural formation, water
**Architecture**	No vegetation, abiogenic landscape, artificially constructed, high morphology
**Forest**	With vegetation, biogenic landscape, partly natural growth, tree
**Grassland/Shrub**	With vegetation, biogenic landscape, partly natural growth, grass
**Farmland**	With vegetation, biogenic landscape, artificially dominated, grass

**Fig 5:**
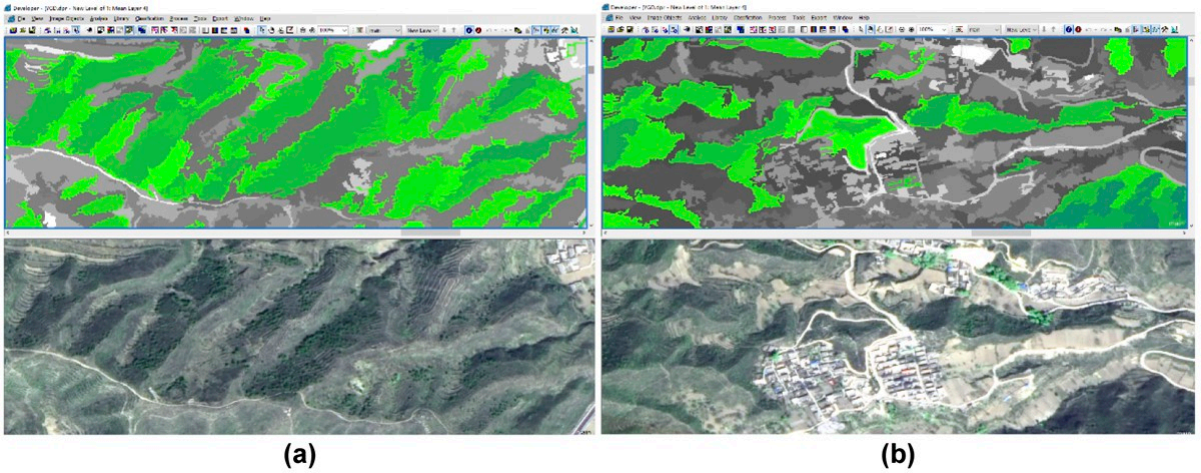
Effects of scenic resource identification after segmentation (using forest as an example). (a) Mountain and (b) residential areas (Republished from [https://directory.eoportal.org/web/eoportal/satellite-missions/g/gaofen-2] under a CC BY license, with permission from [Hebei Zhongke Sino Star Information Technology Co., Ltd], original copyright [2018]).

The established recognition rules were followed to classify the five types of scenic resources: forest, grassland/shrub, farmland, architecture, and water. The resulting scenic resource recognition effect after segmentation is shown in Fig 5. The features of mountain shadows in RS images are similar to those of trees and water. Thus, to improve the classification accuracy of forests and water during identification, the images were revised with artificial visual interpretation.

### Methods for evaluating the classification accuracy of different landform units

Often, differences occur between the classification results of RS images and the actual situation; thus, an accurate evaluation of the classification results is needed to determine the accuracy and reliability of the classification. In this study, a confusion matrix was used for accuracy evaluation [42], and its indices are listed in Table 2. The kappa coefficient was calculated in the range of 0–1 [43], and the values were generally divided into five groups to indicate the level of consistency: 0.0–0.2 indicates slight consistency; 0.2–0.4 indicates fair consistency; 0.4–0.6 indicates moderate consistency; 0.6–0.8 indicates substantial consistency; and 0.8–1 indicates almost perfect consistency [44].

**Table 2:** Evaluation indicators for classification accuracy.

Accuracy evaluation index	Calculation formula	Description
Overall Accuracy (OA)	$p_{c}=\sum_{k=1}^{n}p_{kk}/p$	Total number of correct classifications divided by the number of references.
Producer’s Accuracy (Prod. Acc)	*p_ij_* = *p_ii_*/*p*_*i*+_	Percentage of reference data that are correctly classified.
User’s Accuracy (User. Acc)	*p_ji_* = *p_ii_*/*p*_+*j*_	The number of correct classifications in the same class as a percentage of the total number in the class, indicating the probability that a classified pixel can truly represent the class.
Kappa	$K=\frac{p\times \sum_{k=1}^{n}p_{kk}-\sum_{i=1}^{n}\left(p_{+i}\times p_{i+}\right)}{p^{2}-\sum_{i=1}^{n}\left(p_{+i}\times p_{i+}\right)}$	A statistical value of classification accuracy ranging from 0–1, showing how much better this classification method used is compared to randomly assigning each pixel to any class.

Systematic sampling was then used to extract the classification results corresponding to each classification method to verify and evaluate the accuracy and differences between pixel-based and object-oriented classification. This was carried out based on the forest resource planning and design survey data of Yesanpo National Park in 2018 combined with the vector data of land use raster maps and field surveys in Yesanpo National Park 2017–2030 master plan; from this, the survey data on forest resource planning and design were newly revised and merged in terms of resource types, and the attributes of the survey data on forest resource planning and design were merged into the five categories used in this study (forest, grassland/shrub, farmland, architecture, and water). The actual scenic resource type corresponding to each sampling point was then extracted from the corrected survey data on forest resource planning and design using the ArcGIS multi-value point extraction function as the true value test sample, with the sampling interval set to 100 × 100 m to effectively represent all classifications in the national park. In total, 27,691 sampling points were obtained, including 12,329 for forests, 12,866 for grassland/shrubs, 1,496 for farmland, 804 for architecture, and 196 for water. Within the target scenic spots, Baili Gorge had 15,345 sample points, Longmen Tianguan had 6,575, and Yugu Cave had 5,771. All three scenic spots contained the above five types of scenic resources, and the number of samples for the minimum scenic resource type was above 10.

## Results

### Accuracy evaluation of object-oriented scenic resource classification

#### Accuracy evaluation of object-oriented classification of Baili Gorge

**Fig 6:**
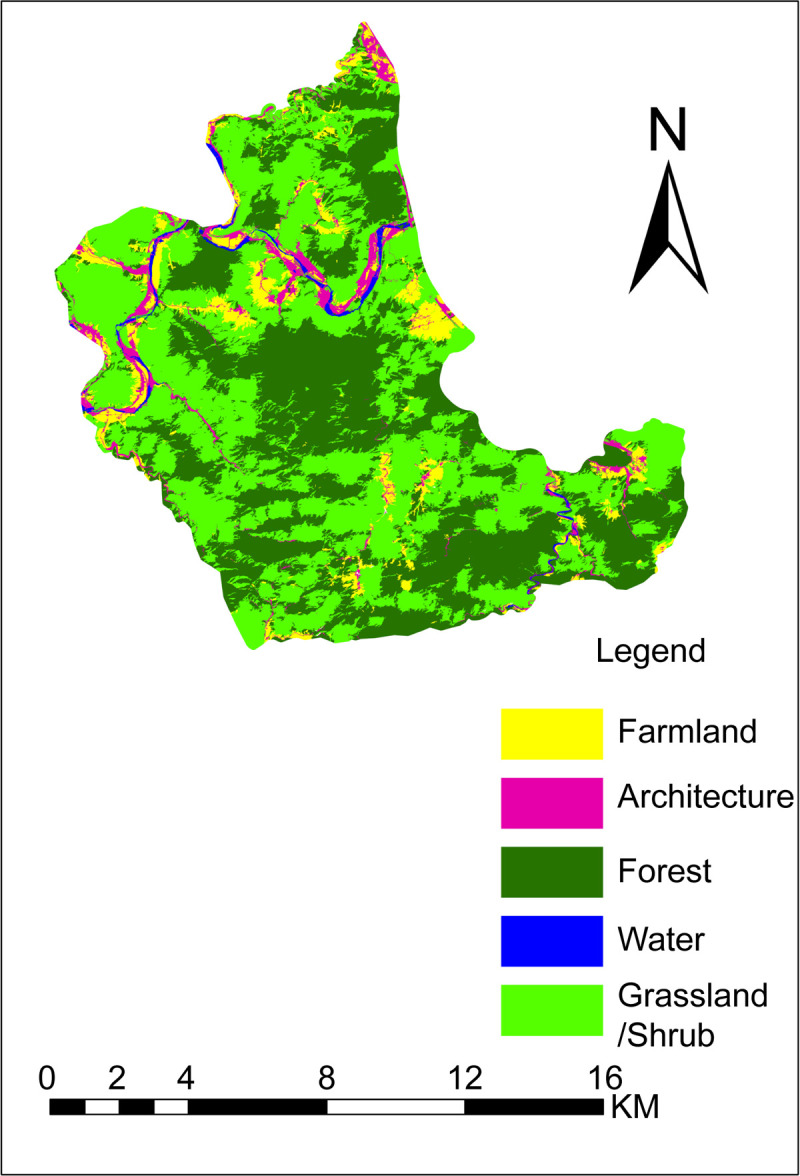
Object-oriented classification of Baili Gorge (Republished from [https://directory.eoportal.org/web/eoportal/satellite-missions/g/gaofen-2] under a CC BY license, with permission from [Hebei Zhongke Sino Star Information Technology Co., Ltd], original copyright [2018]).

The results of the object-oriented classification of Baili Gorge are shown in Fig 6, the corresponding confusion matrix is shown in Table 3, and the overall classification accuracy of the area is shown in Table 4.

**Table 3:** Confusion matrix for the object-oriented classification of Baili Gorge.

Type	Forest	Grassland/shrub	Farmland	Architecture	Water	Sample point
**Forest**	6365	698	296	51	0	7410
**Grassland/shrub**	342	5893	211	107	18	6571
**Farmland**	42	126	536	97	6	807
**Architecture**	6	29	51	313	1	400
**Water**	8	12	16	1	120	157
**Sample point**	6763	6758	1110	569	145	15345

**Table 4:** Accuracy of the object-oriented classification of Baili Gorge.

Type	Forest	Grassland/shrub	Farmland	Architecture	Water	Prod. Acc	Omission
**Forest**	85.90	9.42	3.99	0.69	0.00	85.90	14.10
**Grassland/shrub**	5.20	89.68	3.21	1.63	0.27	89.68	10.32
**Farmland**	5.20	15.61	66.42	12.02	0.74	66.42	33.58
**Architecture**	1.50	7.25	12.75	78.25	0.25	78.25	21.75
**Water**	5.10	7.64	10.19	0.64	76.43	76.43	23.57
**User**	94.12	87.20	48.29	55.01	82.76	OA	86.20
**Commission**	5.88	12.80	51.71	44.99	17.24	Kappa	0.77

Note: Values in the table are percentages (%), except for the kappa coefficient.

Table 4 shows that the overall accuracy of object-oriented classification of Baili Gorge is high (86.20%). The kappa coefficient was 0.77 (between 0.6 and 0.8), indicating a high degree of consistency. The classification described five types of scenic resources:

1. Forest: Primarily classified as grass/shrub (9.42%) and farmland (3.99%) because it can be easily confused with grassland/shrub. Several scattered forests around the farmland exist and show small differences; therefore, these forests can easily be misclassified. The final classification area of forests was smaller than the actual area with a mapping accuracy of 85.90%.
2. Grassland/shrub: Primarily misclassified as forest (5.20%), farmland (3.21%), and architecture (1.63%) because of mixing of some grassland/shrub areas with forests. Crops from some farmlands are similar to those from grassland/shrubs and, therefore, can be easily misclassified. In addition, as the architecture in this area mainly comprises rural settlements and tourist facilities, several vegetation areas comprising grass/shrubs and architecture can be misclassified. The final classification area of grassland/shrub was larger than the actual area with a mapping accuracy of 89.68%.
3. Farmland: Primarily misclassified as grass/shrub (15.61%), architecture (12.02%), and forest (5.20%) because the RS data were acquired in April 2018. The characteristics of crops planted in the farmlands during spring are similar to those of plants in grasslands/shrubs, and, therefore, can be easily misclassified. Several farmlands exist around few architectural structures, leading to misclassification. In addition, there are several scattered forests around the farmland, and there are minor differences between the farmland and the forests. The classified area of farmland was larger than the actual area with a mapping accuracy of 66.42%.
4. Architecture: Misclassified into farmland (12.75%), grassland/shrub (7.25%), and forest (1.50%) because of the presence of several farmlands, grasslands/shrubs, and forests around the architecture. The final architecture classification area was larger than the actual area with a mapping accuracy of 78.25%.
5. Water: Classified as farmland (10.19%), grassland/shrub (7.64%), and forest (5.10%) because pond stems and riverbanks of the water category are easily classified as farmland, grassland/shrub, and forest. The final classification area of water was smaller than the actual area with a mapping accuracy of 76.43%.

#### Accuracy evaluation of object-oriented classification of Longmen Tianguan

**Fig 7:**
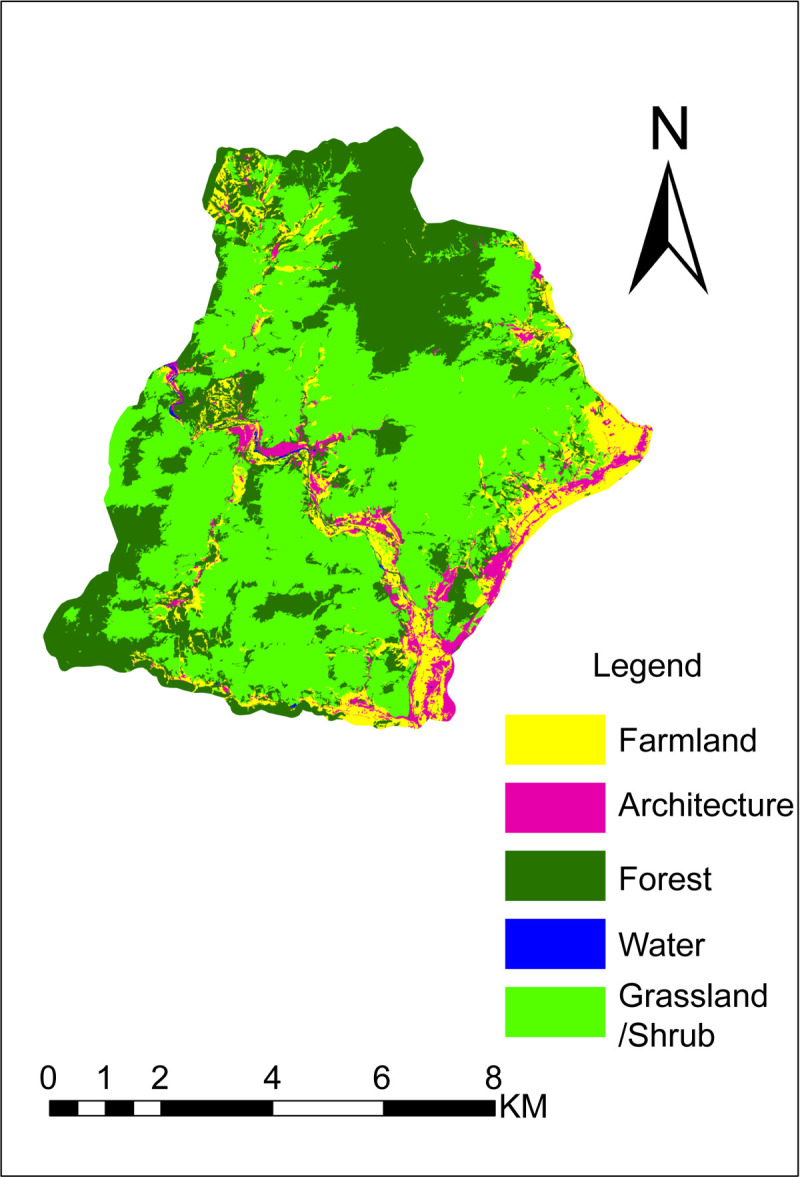
Object-oriented classification of Longmen Tianguan (Republished from [https://directory.eoportal.org/web/eoportal/satellite-missions/g/gaofen-2] under a CC BY license, with permission from [Hebei Zhongke Sino Star Information Technology Co., Ltd], original copyright [2018]).

The results of object-oriented classification are shown in Fig 7, the confusion matrix is shown in Table 5, and the classification accuracy is shown in Table 6.

**Table 5:** Confusion matrix for the object-oriented classification of Longmen Tianguan.

Type	Forest	Grassland/shrub	Farmland	Architecture	Water	Sample point
**Forest**	2131	272	204	31	1	2639
**Grassland/shrub**	130	3123	95	32	0	3380
**Farmland**	15	27	232	40	0	314
**Architecture**	10	14	16	190	0	230
**Water**	1	0	0	2	9	12
**Sample point**	2287	3436	547	295	10	6575

**Table 6:** Accuracy of the object-oriented classification of Longmen Tianguan.

Type	Forest	Grassland/shrub	Farmland	Architecture	Water	Prod. Acc	Omission
**Forest**	80.75	10.31	7.73	1.17	0.04	80.75	19.25
**Grassland/shrub**	3.85	92.40	2.81	0.95	0.00	92.40	7.60
**Farmland**	4.78	8.60	73.89	12.74	0.00	73.89	26.11
**Architecture**	4.35	6.09	6.96	82.61	0.00	82.61	17.39
**Water**	8.33	0.00	0.00	16.67	75.00	75.00	25.00
**User**	93.18	90.89	42.41	64.41	90.00	OA	86.46
**Commission**	6.82	9.11	57.59	35.59	10.00	Kappa	0.77

Note: Values in the table are percentages (%), except for the kappa coefficient.

Table 6 shows that the overall accuracy of object-oriented classification of Longmen Tianguan is high (86.46%). The kappa coefficient was 0.77 (between 0.6 and 0.8), indicating a high degree of consistency. The classification described in five types of scenic resources:

1. Forest: Mainly misclassified as grass/shrub (10.31%), farmland (7.73%), and architecture (1.17%) because it can be easily confused with grassland/shrub. Furthermore, the presence of several scattered forests around farmlands and architectural structures, having small differences, results in misclassification. The final classification area of forests was smaller than the actual area with a mapping accuracy of 80.75%.
2. Grassland/shrub: Mainly misclassified as forest (3.85%), farmland (2.81%), and architecture (0.95%) because some grassland/shrub areas are mixed with forest, which causes misclassification. The characteristics of the crops planted in the farmland during spring are similar to those in grassland/shrubs and can, therefore, be misclassified. In addition, certain grasses/shrubs are mistakenly classified as architecture because the architecture in this area mainly comprises rural settlements and tourist facilities; additionally, numerous vegetation areas occur around some architectural structures, thereby causing misclassification. The final classification area of grassland/shrub was larger than the actual area with a mapping accuracy of 92.40%.
3. Farmland: Mainly misclassified as architecture (12.74%), grassland/shrub (8.60%), and forest (4.78%) because of the presence of numerous farmlands around some architectural structures that could cause misclassification. The characteristics of crops planted in farmlands during spring are similar to those of plants in grasslands/shrubs and can, therefore, be easily misclassified. In addition, several scattered forests having small differences exist around farmlands, facilitating misclassification. The final classification area of farmlands was larger than the actual area with a mapping accuracy of 73.89%.
4. Architecture: Primarily classified as farmland (6.96%), grassland/shrub (6.09%), and forest (4.35%) because there are several farmlands, grasslands/shrubs, and forests around the existing architecture. The final architecture classification area was larger than the actual area with a mapping accuracy of 82.61%.
5. Water: Erroneously divided into architecture (16.67%) and forest (8.33%) because pond stems and riverbanks are divided into architecture and forest. Because the RS data were acquired during the dry season (April), some river beaches are mistakenly classified as architecture. The final classification area of the water was smaller than the actual area with a mapping accuracy of 75.00%.

### Accuracy evaluation of object-oriented classification of Yugu Cave

**Fig 8:**
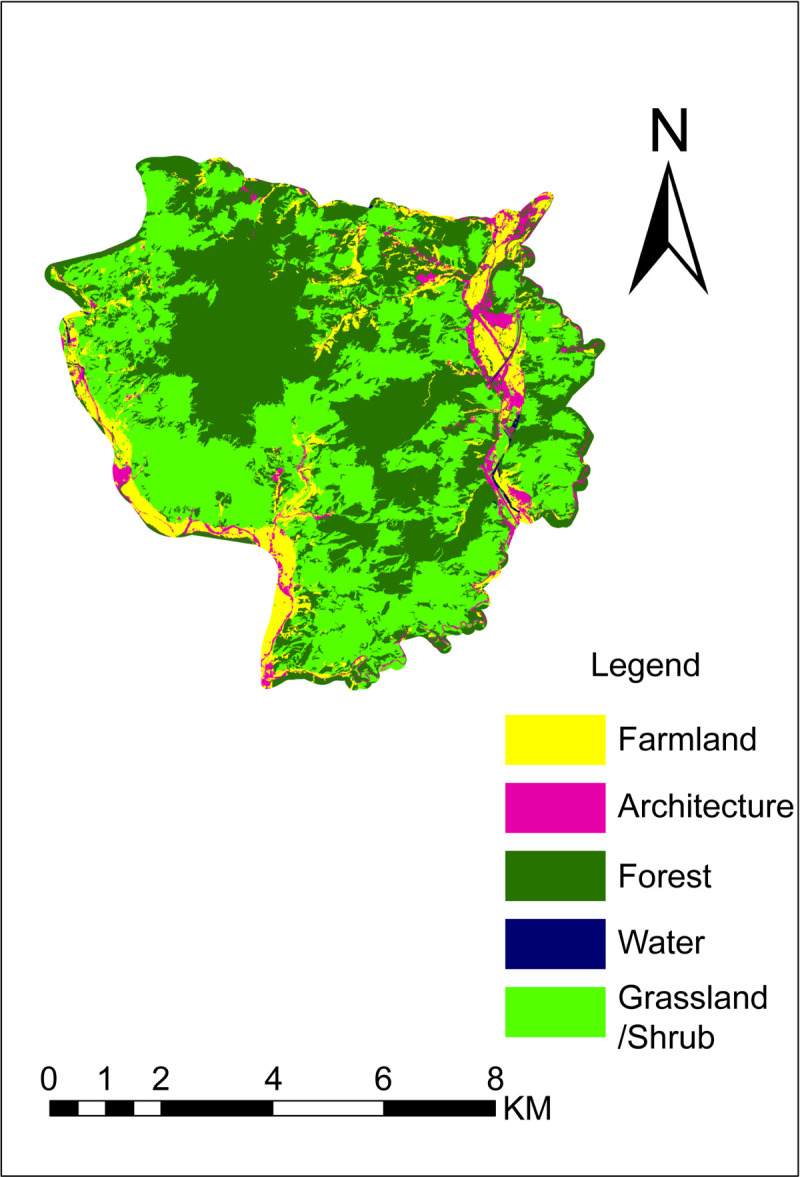
Object-oriented classification of Yugu Cave (Republished from [https://directory.eoportal.org/web/eoportal/satellite-missions/g/gaofen-2] under a CC BY license, with permission from [Hebei Zhongke Sino Star Information Technology Co., Ltd], original copyright [2018]).

The results of object-oriented classification are shown in Fig 8, the confusion matrix is shown in Table 7, and the classification accuracy is shown in Table 8.

**Table 7:** Confusion matrix for object-oriented classification of Yugu Cave.

Type	Forest	Grassland/shrub	Farmland	Architecture	Water	Sample point
**Forest**	2134	91	38	16	1	2,280
**Grassland/shrub**	183	2,609	91	32	0	2,915
**Farmland**	38	19	279	39	0	375
**Architecture**	34	15	22	103	0	174
**Water**	1	0	2	8	16	27
**Sample point**	2,390	2,734	432	198	17	5,771

**Table 8:** Accuracy of object-oriented classification of Yugu Cave.

Type	Forest	Grassland/shrub	Farmland	Architecture	Water	Prod. Acc	Omission
Forest	93.60	3.99	1.67	0.70	0.04	93.60	6.40
Grassland/shrub	6.28	89.50	3.12	1.10	0.00	89.50	10.50
Farmland	10.13	5.07	74.40	10.40	0.00	74.40	25.60
Architecture	19.54	8.62	12.64	59.20	0.00	59.20	40.80
Water	3.70	0.00	7.41	29.63	59.26	59.26	40.74
User	89.29	95.43	64.58	52.02	94.12	Overall accuracy	89.08
Commission	10.71	4.57	35.42	47.98	5.88	Kappa factor	0.82

Note: Values in the table are percentages (%), except for the kappa coefficient.

Table 8 shows that the overall accuracy of object-oriented classification of Yugu Cave is high (89.08%). The kappa coefficient was 0.82 (between 0.8 and 1), indicating almost perfect consistency. The classification system described five types of scenic resources:

1. Forest: Classified as grass/shrub (3.99%) and farmland (1.67%) because it can be easily confused with grassland/shrub. Furthermore, several scattered forests having small differences exist around the farmlands, thereby causing misclassification. The final classification area of forests was larger than the actual area with a mapping accuracy of 93.60%.
2. Grassland/shrub: Misclassified as forest (6.28%), farmland (3.12%), and architecture (1.10%) because some grassland/shrub areas are mixed with forests, thereby causing misclassification. The characteristics of crops planted in the farmlands during spring are similar to those of the plants in grasslands/shrubs, thereby causing them to be easily misclassified. Some grasses/shrubs are mistakenly classified as architecture because the architecture in this area mainly comprises rural settlements and tourist facilities; additionally, numerous vegetation areas around some architectural structures result in easy misclassification. The final classification area of grassland/shrub was smaller than the actual area with a mapping accuracy of 89.50%.
3. Farmland: Misclassified as architecture (10.40%), forest (10.13%), and grassland/shrub (5.07%) because of the presence of numerous farmlands around some architectural structures that can be easily misclassified. Furthermore, the RS data were acquired in April 2018. The characteristics of crops planted in the farmlands during spring are similar to those in grasslands/shrubs and can, therefore, be easily misclassified. In addition, several scattered forests having small differences exist around the farmland, resulting in their misclassification. The final classification area of farmlands was larger than the actual area with a mapping accuracy of 74.40%.
4. Architecture: Misclassified as forest (19.54%), farmland (12.64%), and grassland/shrub (8.61%) because of the presence of several farmlands, grasslands/shrubs, and forests around the existing architecture that are easily misclassified. The final architecture classification area was larger than the actual area with a mapping accuracy of 59.20%.
5. Water: Erroneously divided into architecture (29.63%), farmland (7.41%), and forest (3.70%) as pond stems and river embankments are easily divided into architecture, farmlands, and forest. Because the RS data were acquired during the dry season (April), some river beaches are mistakenly classified as architecture. The final classification area of the water was smaller than the actual area with a mapping accuracy of 59.26%.

### Evaluation of pixel-based and object-oriented classification methods

**Fig 9:**
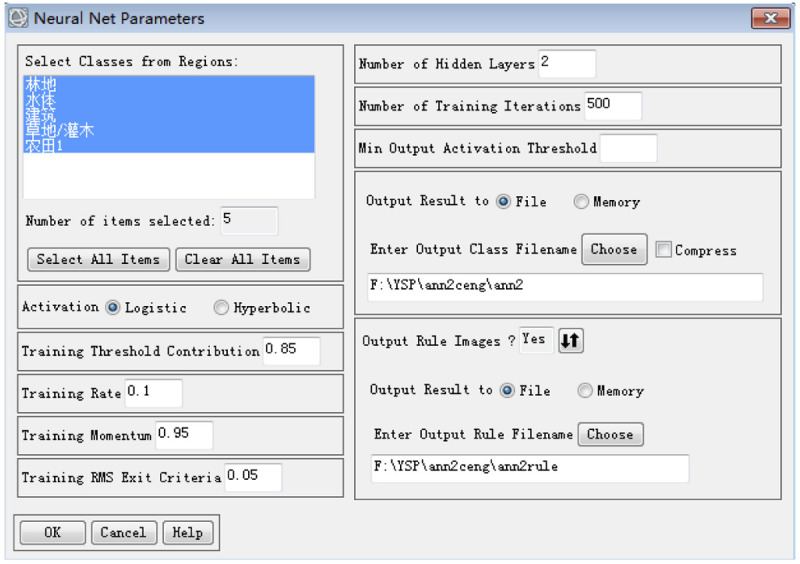
NN parameter settings.

**Fig 10:**
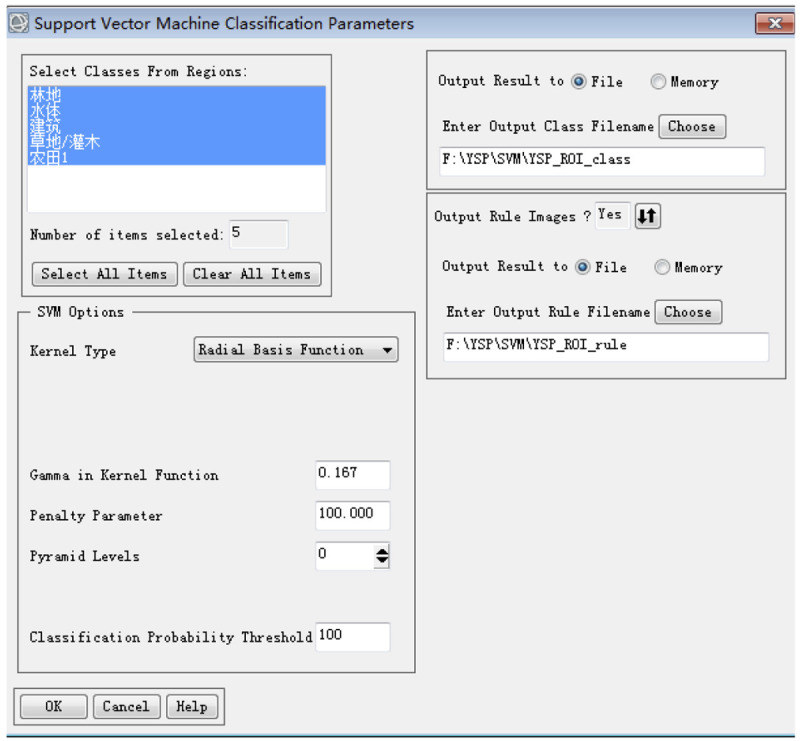
SVM parameter settings.

In this study, the evaluation samples were selected by systematic sampling, which is more flexible and objective than the traditional method of obtaining evaluation samples. This method was used to calculate the overall accuracy and kappa coefficient of three pixel-based classification methods (MLC, NN, and SVM); the findings for each pixel-based method were compared with those of the object-oriented classification method. The results are shown in Table 9. The parameters of NN and SVM are shown in Figs 9 and 10.

**Table 9:** Accuracy evaluation of pixel-based and object-oriented classifications.

Scenic area	Classification	Overall accuracy (%)	Kappa
**Baili Gorge**	MLC	79.21	0.65
	NN	77.54	0.62
	SVM	78.19	0.63
	Object-oriented classification	86.20	0.77
**Longmen Tianguan**	MLC	76.99	0.59
	NN	78.66	0.62
	SVM	74.97	0.57
	Object-oriented classification	86.46	0.77
**Yugu Cave**	MLC	77.84	0.62
	NN	77.81	0.62
	SVM	77.92	0.62
	Object-oriented classification	89.08	0.82

In summary, the overall accuracy of the object-oriented classification method ranged from 86.20–89.08% and the kappa coefficient ranged from 0.77–0.82, showing a good classification result. The main reason for this outcome is that the object-oriented method is able to avoid the excessive confusion that typically occurs between the following combinations of resource types: forest and grassland/shrub; grassland/shrub and farmland; and water, forest, and grassland/shrub. For this method, the difference between forest and grassland/shrub in spectral features was not significant. However, for the pixel-based classification method, which is mainly based on spectral features, the forest and grassland/shrub had little difference and were classified into one category, resulting in a low accuracy of forest classification. The same problem also occurred between grassland/shrub and farmland as their spectral features were similar; thus, it was difficult to make a fine distinction between them. In addition, because of the existence of mountain shadows, the features within the shaded regions were similar to those of water; thus, the pixels of the shaded part were falsely classified as water.

## Discussion and conclusion

To select the most suitable classification method for scenic resource identification, four classification methods were considered, object-oriented and three pixel-based (MLC, NN, SVM), to identify the scenic resources of three representative landforms in Yesanpo National Park from GF-2 satellite images. The accuracy of the recognition effect of the four classification methods was evaluated using a systematic sampling method. The evaluation results were as follows: 1) Baili Gorge (erosional alluvial landform), object-oriented classification (86.20%) > MLC (79.21%) > SVM (78.19%) > NN (77.54%); 2) Longmen Tianguan (granite fracture structure canyon landform), object-oriented classification (86.46%) > NN (78.66%) > MLC (76.99%) > SVM (74.97%); and 3) Yugu Cave (karst-cave spring landform), object-oriented classification (89.08%) > SVM (77.92%) > MLC (77.84%) > NN (77.81%). The results showed that the scenic resources of the three landforms met the requirements of classification accuracy using MLC, NN, SVM, and object-oriented classification and that object-oriented classification had higher accuracy and was more suitable for the systematic and high-precision identification of scenic resources in GF-2 images. The method for scenic resource identification based on GF-2 images was finalized as follows. First, a decision tree model was used to construct a scenic resource identification system, which was in turn used as classification criteria for the identification of five types of scenic resources: forest, grassland/shrub, farmland, architecture, and water. Land use raster map and forest resource planning and design survey data were then incorporated to construct a scenic resource database in GIS and the spatial analysis functions of GIS were used to refine the classification and identification of scenic resources [45]. This approach greatly increases the precision of scenic resource identification and completely utilizes raster and vector data such as GF-2 images, digital elevation models, and forest resource planning and design survey data. The advent of this method will aid in the digitalization and systemization of scenic resource identification.

Pixel-based classification primarily analyzes the spectral features of pixels during classification. Conversely, high-resolution RS images usually contain few bands and limited spectral information; thus, they are not only based on the spectral information of pixels but can also be combined with spatial feature information such as shape and texture when classifying high-resolution images. Because of this, pixel-based classification fails to fully utilize the rich spatial data of high-resolution images, which leads to wastage of spatial data resources to a certain extent, thereby lowering the classification accuracy and reducing the method’s effectiveness in terms of classification and recognition [46].

In comparison, the object-oriented classification method overcomes some defects of pixel-based classification because it combines the spectral, textural, and morphological characteristics of scenic resources in the process of classification, groups the pixels with the same characteristics into a patch by means of clustering, and then selects the patch as a feature for classification according to the characteristics of scenic resources. This process avoids the “salt-and-pepper-effect” results of the pixel-based classification method, increasing the differences between the resource combinations of forest and grassland/shrub; grassland/shrub and farmland; and water, forest, and grassland/shrub, effectively improving the overall classification accuracy. Moreover, when object-oriented classification is used to process high-resolution RS images, this method can compensate for the disadvantage of unstable spectral recognition in such images and fully utilize the advantage of the rich spatial data of that image type.

However, it should be noted that although the object-oriented classification method improves the classification accuracy, it does not completely address all the problems pertaining to pixel-based classification methods. For example, more restrictive factors need to be added to the classification to improve the separability among scenic resources, which is the priority of future research. Moreover, the object-oriented classification method has a higher requirement for operators, such that operators must not only master the geometric information, structural information, and spectral information characteristics and establish corresponding classification rules of the scenic resources to be identified but also establish classification rules for geometric, structural, and spectral information and select effective combinations of feature parameters to improve the classification accuracy. As a result, the actual time needed for classification is longer than that needed to carry out pixel-based classification.

Therefore, it can be concluded that the scope of application of pixel-based and object-oriented classification methods is different. Hence, these should be used according to the actual situation and needs of scenic resource surveys. If multi-band, hyperspectral RS images are used, or if rapid screening of scenic resources is required for RS images, pixel-based classification can be used and then combined with historical information to identify key survey areas and field survey routes in GIS. Using this, detailed identification can be carried out using field survey data combined with visual interpretation. Alternately, for the systematic and high-precision identification of scenic resources in high-resolution RS images, object-oriented classification may be used. The scenic resource identification system proposed in this study can be employed to establish classification rules, and effective feature parameter combinations can be selected to improve classification accuracy. The GF-2 images of Baili Gorge, Longmen Tianguan, and Yugu Cave scenery areas are suitable for the object-oriented classification method, which has the highest classification accuracy and is the most efficient.

In the past few years, the use of high-resolution RS images as data sources and that of 3S technology to classify scenic resources have attracted the attention of the geographic information and landscape architecture research communities. The classification of scenic resources can be combined with 3S technology to identify and highlight the spatial combination information of scenic resources. This study proposes a scientific and effective method, using GF-2 images as the data source, as well as pixel-based (MLC, NN, and SVM) and object-oriented classification methods, to identify scenic resources and introduces systematic sampling methods to evaluate classification accuracy. The results show that the object-oriented classification has the highest accuracy in identifying scenic resources among the four classification methods. Because of the difference between pixel-based and object-oriented classification principles, the scientific evaluation of its classification accuracy has consistently been called into question in geographic information research and application. This study proposes a systematic sampling method that uses the same evaluation index and accuracy evaluation sample in ArcGIS to evaluate scenic resources using pixel-based and object-oriented classification accuracy, which effectively reduces the difference and accuracy evaluation of recognition software (ENVI, eCognition). Hence, this evaluation method can effectively improve the accuracy and objectivity of evaluations based on pixel-based and object-oriented classification accuracy.

The scenic resource identification system constructed in this study is based on a decision tree model and can be used to establish object-oriented classification guidelines. Moreover, it is suitable for the efficient identification of five types of scenic resources, namely, forest, grassland/shrubland, farmland, architecture, and water, thus, the classification accuracy and speed can be effectively improved.

After field investigation and verification of scenic resources in Yesanpo National Park, it was found that the scenic resource data identified by this method better reflect the actual situation of the resources in the park; therefore, this method is suitable for the future application of scenic resource identification in natural national parks. This method show promise for building a scenic resource GIS database and more efficiently managing scenic resource text, charts, and other data. In the future, a variety of spatial analysis tools can be used for the spatial analysis and evaluation of scenic resource data. Therefore, promoting the standardization and informatization of scenic resource identification and evaluation will help improve their accuracy and efficiency [47], effectively providing planners and managers with relevant data for development and environmental protection decision-making. Scientific planning of the district and effective management of the related national park can also provide basic data and decision-making support.

One limitation of the current study was that the analyzed data were specific to the month of April. Thus, we need to select and assess the national park RS data for all four seasons to compare and identify changes in the combination of scenic resources in different seasons, thereby fully exploring the value of scenic resources. With the continuous development of 3S technology, we will continue to work toward introducing improved technologies, including higher resolution RS images and drone tilt photography, to the scenic resource identification system to meet the needs of national parks with unique planning and management needs.
